# A lightweight thermally insulating and moisture-stable composite made of hollow silica particles†

**DOI:** 10.1039/d2ra01561g

**Published:** 2022-05-20

**Authors:** Jaswinder Sharma, Georgios Polizos, Charl J. Jafta, Yaocai Bai, Diana Hun, Xiang Lyu

**Affiliations:** Electrification and Energy Infrastructure Division, Oak Ridge National Laboratory Oak Ridge TN 37830 USA sharmajk@ornl.gov +1-865-241-2333; Building Technologies Research and Integration Center, Oak Ridge National Laboratory Oak Ridge TN 37830 USA

## Abstract

Thermal insulation materials are highly desirable for several applications ranging from building envelopes to thermal energy storage systems. A new type of low-cost insulation material called hollow silica particles (HSPs) was recently reported. The present work presents an HSP-based stand-alone composite that has very low thermal conductivity and is highly stable to moisture.

## Abstract 2

A very low thermal conductivity and moisture stable composite made of hollow silica particles and cellulose fibers.

## Graphical abstract

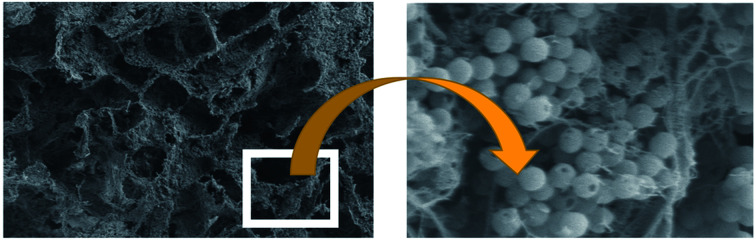

## Introduction

Thermal insulation materials are highly desirable for insulation in refrigerators, building envelopes, cryogenic storage systems, high-temperature fuel cells, thermal energy storage systems, combined heat and power systems, and heat exchangers.^1–8^ Conventional insulation materials (*e.g.*, mineral wool, glass fiber, cellulose fibers, and expanded polystyrene) have high thermal conductivities (ranging from 0.030 to 0.040 W m^−1^ K^−1^).^1,9^ Therefore, to achieve better thermal insulation, a thick layer of these materials is required, which results in high material, transportation, and disposal costs. Aerogels are a state-of-the-art insulation material with thermal conductivities ranging from 0.012 to 0.020 W m^−1^ K^−1^.^10–12^ Aerogels provide the best thermal insulation. A thin layer of aerogel can provide the same level of insulation that a thick layer of conventional insulation material provides. However, aerogels' high cost, fragility, and lack of scalability hinder their commercial use.^10–13^ A new type of insulation material called hollow silica particles (HSPs), which have a thermal conductivity of 0.02–0.03 W m^−1^ K^−1^, was recently reported.^14–19^

HSPs can be synthesized by several strategies, such as by using polymer reverse microemulsions, micelles, polymer (*e.g.*, polystyrene [PS]) particles, inorganic (*e.g.*, calcium carbonate, carbon, hydroxy apatite) particles, and bacteria as templates or by etching the solid silica particles.^14–31^ We recently demonstrated that HSPs can be synthesized very inexpensively by recycling the solvents.^32^ HSPs can provide very high thermal insulation at a lower cost; however, because HSPs are a powder that is usually in the nano-micrometer size range, they are a health hazard, difficult to handle and transport, and can be messy to use.^1,33,34^ For example, it is difficult to fill HSPs in a wall. Therefore, HSPs cannot be used as a stand-alone thermal insulation and are generally mixed with other materials to make a composite thermal insulation material. Mixing HSPs in polymer matrices has been extensively explored.^35–37^ Ernawati *et al.* showed that mixing HSPs with polyethersulfone polymer results in a composite thin (35 μm) film with a thermal conductivity of ∼0.03 W m^−1^ K^−1^, whereas films made of polyethersulfone alone had a thermal conductivity 0.09 W m^−1^ K^−1^.^37^ Nevertheless, mixing HSPs with polymers generally provides composites with thermal conductivities ranging from 0.03 to 0.08 W m^−1^ K^−1^, which are very high compared with other insulation materials and HSPs itself. Because matrix polymers have high thermal conductivities (∼0.15–0.30 W m^−1^ K^−1^), the overall thermal conductivity of HSPs-polymer composites is lower than the polymer but higher than the HSPs. Thus, the original goal of using HSPs as thermal insulation material has become compromised, and HSPs have become only an additive to lower the thermal conductivity of other materials.^35–37^

The present work presents an HSPs-based stand-alone composite that has very low thermal conductivity and is highly stable to moisture. The composite is made by freeze–drying the mixture of HSPs with cellulose fibers (CFs) and carbon black (CB), and it is made highly hydrophobic without compromising its structural integrity and thermal conductivity and without using an expensive hydrophobization strategy.

## Experimental

### Composite fabrication

HSPs with an average diameter of 300 nm were synthesized by using a previously reported method (details ESI†).^30,32^ CFs (diameter: 50–100 nm; 3% solid content, from University of Maine Process Development Center) were mixed in water (4 mg mL^−1^) by homogenizing with a homogenizer (IKA-T18 digital Ultra Turrax) at 15 000 rpm for 8 h before mixing with HSPs. HSPs and a CF-based composite was prepared by mixing 80 wt% HSPs with 19.5 wt% CF in water. Additionally, 0.5 wt% of CB (C-NERGY SUPER C45 from Imerys ≈ 50 nm particle size) was added to the mixture. The mixture was mixed by stirring on a magnetic stir plate at 200 rpm for 10 min. Then, the mixture was added into different molds and placed in a freezer at −15 °C overnight. The frozen mixture was dried *via* a freeze–drying process (labconco freeze dryer: catalogue# 7740020).

### SEM imaging

Scanning electron microscopy (SEM) imaging was performed by using Merlin 200 SEM at the Center for Nanophase Materials Sciences user facility at Oak Ridge National Laboratory. All samples were deposited on silicon wafers, which were then attached to the SEM stubs *via* carbon tapes.

### Composite hydrophobization

**Fig. 1:**
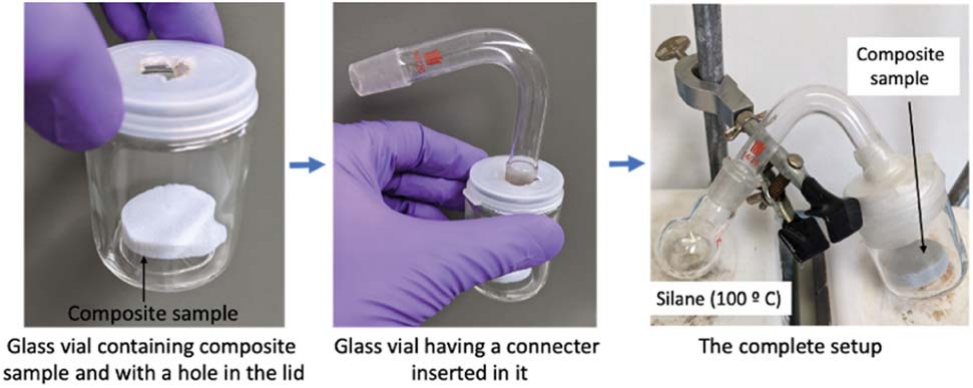
Steps used to make a vapor phase hydrophobic coating on the composite sample.

The composite sample was placed inside a glass vial. A round-bottomed flask containing hydrophobic silane (trimethoxymethylsilane: 1 cm^3^) was connected with this glass vial by making a hole in the lid. The lid hole was tightly closed by using paraffin film. The flask was heated to 100 °C to vaporize the silane. The composite sample was flipped every 30 m to ensure that each side was uniformly coated.^30^Fig. 1 shows the different steps of the hydrophobization process.

### Thermal conductivity measurements

Thermal conductivity measurements were performed using a Transient Plane Source (2500S) instrument. Thermal conductivity values reported were measured by putting 50 g weight on the loose particles and composite pieces in order to get better contact of composite with the sensor (more details in ESI†).

### Moisture adsorption measurements

Moisture adsorption measurements were performed by placing the hydrophilic and hydrophobic samples in a homemade humidity chamber and then heating water in a closed container. The relative humidity (RH) was ∼90%. The samples were placed in this chamber for 2 weeks. The sample weight was measured before and after being placed in the humidity chamber.

### Surface area and pore size distribution measurement

The specific surface area and pore size distribution measurements were collected from a N_2_ isothermal adsorption experiment by Brunauer–Emmett–Teller (BET) (Nova Touch) with a density-functional theory model.

### FTIR analysis

The surface groups were studied by Fourier-transform infrared (FTIR) spectroscopy (Bruker).

### Contact angle measurements

Contact angle measurements were performed *via* an optical tensiometer (Biolin Scientific). For each contact angle measurement, a 5 μL droplet of de-ionized water was dispensed onto the sample surface and imaged for 3 s. Contact angles were recorded and extracted by image analysis software to minimize human error associated with droplet detection and analysis.

## Results and discussion

**Fig. 2:**
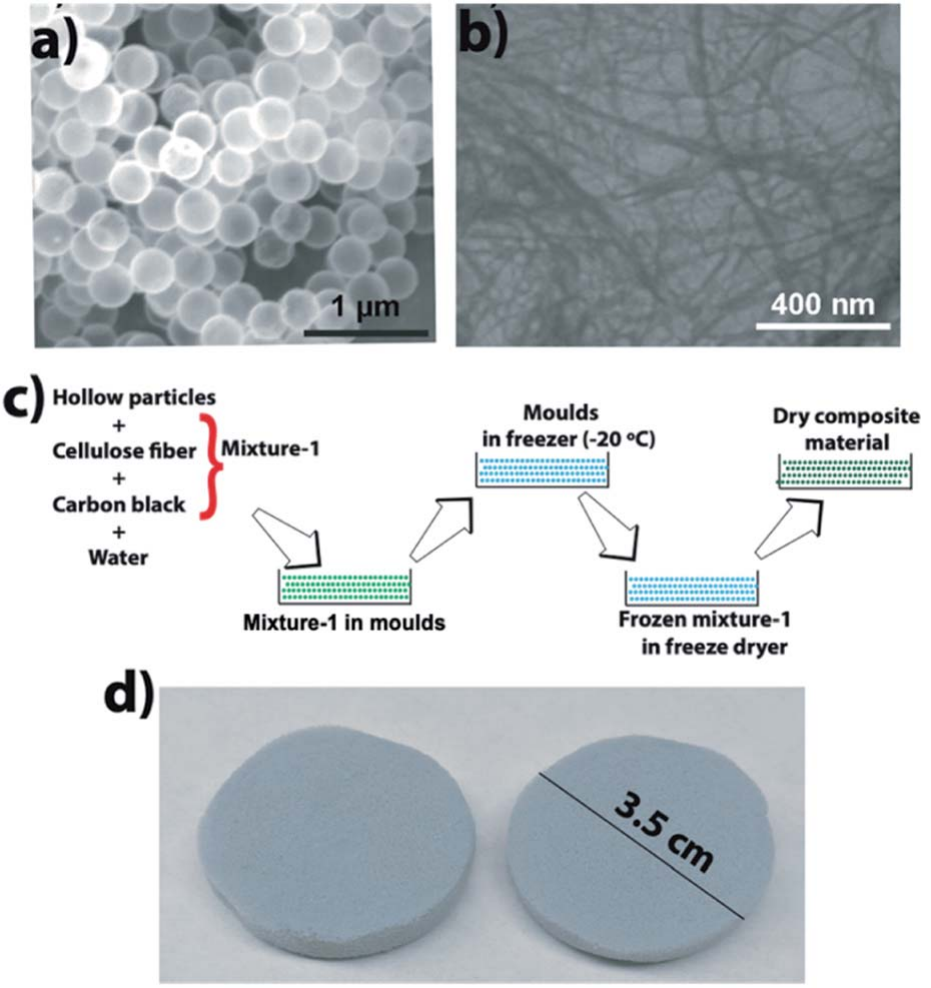
(a) SEM image of HSPs, (b) SEM image of CFs, (c) schematic showing the composite fabrication, and (d) photo of the composite material.

The composite was made by mixing CFs and HSPs in water, then pouring the mixture in molds and removing water *via* a freeze–drying technique. Fig. 2 shows an SEM image of HSPs, an SEM image of CFs, a schematic that shows the composite fabrication, and a photo of fabricated composite material.

The thermal conductivity of the composite was ∼0.025 ± 0.003 W m^−1^ K^−1^, which was lower than both the CFs (0.05 W m^−1^ K^−1^)^1^ and HSPs (0.028 ± 0.002 W m^−1^ K^−1^) alone. This indicates that the composite retains the thermal insulation properties of the HSPs. In all other composites reported to date, the thermal conductivity of the composite is always higher than the thermal conductivity of HSPs.^35–38^

**Fig. 3:**
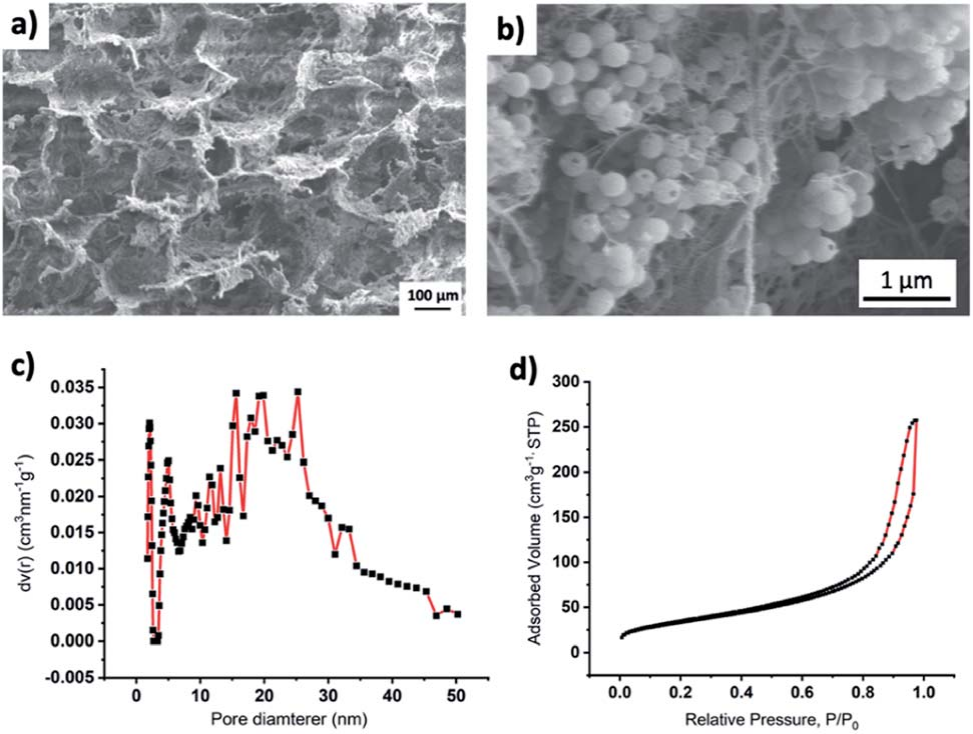
(a) Low magnification SEM image of composite material showing large pores created by sublimation of ice, (b) high magnification SEM image of composite wall showing distribution of CF and HSPs, (c) pore size distribution using the NLDFT adsorption branch model, cylindrical/sphere pores with nitrogen sorption on silica, and (d) nitrogen adsorption–desorption isotherm plots of the composite material.

SEM imaging (Fig. 3a) of the composite material showed larger pores in the range of 10 to 100 μm. Composite density was ∼30 mg cm^−3^. Density was measured by dividing the weight of the composite sample with its volume. Additionally, BET analysis showed that there were only a few mesoscale (2–50 nm) pores (Fig. 3b). The mesopore surface area was 112.6, and the mesopore volume was 0.3885 cm^3^ g^−1^. From SEM images and BET analysis, we assume that the lowered thermal conductivity may have resulted from both the increased air volume (HSPs have a density ≈ 40 mg cm^−3^ while the composite has a density ≈ 30 cm^3^) and increased interfacial contact resistance, *i.e.*, Kapitza resistance.^38^ An increase in interfacial contact resistance is expected due to an increase in the heterogenous interfaces (interfaces between HSPs and CF, between HSPs and CB, and between CF and CB) in the composite while the HSPs have only one type of interface, *i.e.*, HSP-HSP. It has been reported that heterogenous nature of interfaces results in an increase in phonon scattering, and therefore, heat transfer at heterogenous interfaces is less when compared to the heat transfer through the homogenous interfaces where less phonon scattering occurs.^38,39^ Since composite has more heterogenous interfaces than that of the HSPs alone, it is expected that this increased number of heterogenous interfaces also plays a role in lowering the heat transfer through the composite, and thus making it a better insulating material than the HSPs alone.

In one experiment, we added 1.0 cm^3^ of water to 0.2 cm^3^ of HSPs. The total volume was ∼1.0 cm^3^, which indicated that water enters the hollow particle cavity. Water was expected to enter inside the hollow particle cavity because of the hydrophilic (–OH groups on the surface) and porous nature of the HSP shell. Water entering in the HSPs cavity resulted in the hollow particles settling at the bottom of the mixture. Because water enters the hollow particle cavity, the freeze–drying process was expected to break the hollow particles as a result of water expanding during freezing. To check whether the freeze–drying process breaks the particles, we dispersed a small amount of particles in water, froze the mixture, and then freeze–dried them. SEM imaging showed that only a small fraction (∼5–10% by number) of particles was broken during this process. Because only a small fraction of particles broke, a large fraction (≥90%) of particles remains intact and thus contributes to lowering the thermal conductivity of the composite.

**Fig. 4:**
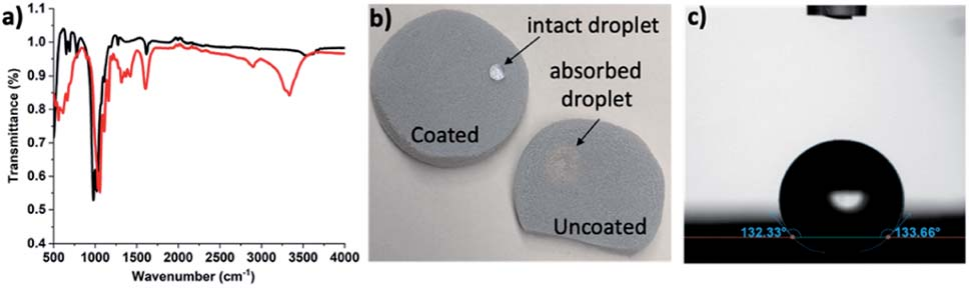
(a) FTIR spectra of coated (black line) and uncoated (red line) composite, (b) a photograph showing an intact water droplet on the coated sample and the absorbed water droplet in the uncoated sample, and (c) the image of a sessile drop on the coated sample used for contact angle measurements.

The end goal of several thermal insulation materials is to be used in real-world applications, such as in a building envelope where moisture is present.^1–3^ A thermal insulation material that is sensitive to moisture is unsuitable for such applications because moisture degrades the material and results in an increase in its thermal conductivity, thus deteriorating its thermal insulation properties.^40^ Generally, the thermal insulation materials are made hydrophobic or water stable by either depositing a hydrophobic silane or hydrophobic polymer coating.^41^ The hydrophobic coatings can be deposited on different materials by using a solution phase approach in which the material is dipped inside a silane solution.^42^ Because our composite is hydrophilic, we coated it with hydrophobic silanes using our indigenously developed low-cost coating technique to enhance its moisture stability without compromising with its thermal insulation properties.^30^ To confirm the successful coating of the composite, FTIR analysis was performed, which clearly showed the disappearance of –OH group peak at ∼3300 in the coated sample when compared with the uncoated sample (Fig. 4a). Similar to the previous reports, we could not see a clear –CH_3_ group peak because of its very low concentration compared with the Si–O–Si network.^43^

To further confirm the hydrophobic nature of the composite, we measured the contact angle of water on the coated and uncoated samples. When a drop of water is placed on an uncoated sample, it is absorbed immediately. Therefore, measuring the contact angle on the uncoated sample was impossible. However, the coated sample showed a contact angle of ∼132° (Fig. 4c), which shows its hydrophobic nature and indicates that the vapor phase coating strategy can be successfully employed for our composite material.

To further confirm the stability of the hydrophobic silane-coated composite in the moisture, we placed the coated and uncoated samples in moisture (RH > 90%) for 2 weeks. The uncoated sample absorbed 15.82% of its original weight in moisture (original weight, 335 mg; weight after placing in 90% RH, 388 mg; amount of water absorbed, 53 mg). However, the coated sample absorbed only 1.81% of its original weight in moisture (original weight, ∼330 mg; weight after placing in 90% RH, 336 mg; amount of water absorbed, 6 mg). The hydrophobic nature of this composite makes it more attractive for thermal insulation applications.

## Conclusions

We demonstrated a strategy for making a thermal insulation composite material using HSPs. Compared with previously reported HSP composites, the reported composite has almost similar or somewhat lower thermal conductivity than HSPs and thus retained the insulation properties of the HSPs. Additionally, we demonstrated that the composite is highly stable to moisture and thus is more suitable for thermal insulation applications. We expect that this work will address the long-standing issue of using HSPs as a thermal insulation material without compromising with their thermal insulation properties.

## Conflicts of interest

There are no conflicts to declare.

## Supplementary Material

RA-012-D2RA01561G-s001
