# Supplementary material for: A lightweight thermally insulating and moisture-stable composite made of hollow silica particles
Source: RSC Adv. 2022 May 20;12(24):15373–7. doi: 10.1039/d2ra01561g (PMC9121439; doi:10.1039/d2ra01561g)
Supplement: RA-012-D2RA01561G-s001 [file RA-012-D2RA01561G-s001.pdf]

Electronic Supplementary Material (ESI) for RSC Advances.
This journal is © The Royal Society of Chemistry 2022

Electronic Supplementary Information

A lightweight thermally insulating and moisture stable composite made of hollow silica particles

Jaswinder Sharma, Georgios Polizos, Charl J. Jafta, Yaocai Bai, Diana Hun, and Xiang Lyu

Chemicals:

Tetraethyl orthosilicate (TEOS), styrene, ammonium hydroxide (28-30%), trimethoxymethylsilane, and isopropanol were purchased from Sigma Aldrich. 2,2'-Azobis(2-methylpropionamidine) dihydrochloride was purchased from fisher scientific.

Polystyrene particle synthesis:

Polystyrene (PS) particles were synthesized by modifying a reported method [1,2]. In a typical synthesis, 100 mL water was heated at 60 °C for 10 minutes, followed by addition of 4 mL of styrene to this hot water while stirring (600 rpm) using a magnetic stir bar. The reaction mixture was allowed to stir for 1 h before adding 2 mL of 160 mg/1 mL of 2,2'-Azobis(2-methylpropionamidine) dihydrochloride. Then the reaction was allowed to stir for 12 h. The as synthesized particles were dried using an air blow drying oven and dried particles were used as templates for the synthesis of HSPs.

Hollow silica particle synthesis:

The HSPs with an average diameter of 300 nm were synthesized by using a previously reported method [1,2]. Briefly, 5 g of polystyrene particles were mixed in an isopropanol/water (75% : 25%) solution. pH of this solution was raised to ≈ 11.2 by adding ammonium hydroxide solution (28-30% solution from Sigma Aldrich). 4.5 mL tetraethyl orthosilicate was added to this solution while stirring to deposit silica around the polystyrene particles. Let the solution stir overnight, and next day core-shell particles were removed by decanting and were dried at 50 °C by using an air blow drying oven. The dried core-shell particles were poured in a crucible and were burned at 600 °C inside a Barnstead Thermolyne 47900 box furnace for 5 h. The burning removed the polystyrene particles and provided the HSPs.

Test for stability of hydrophobic coating to the normal wear tear/handling:

A composite sample prepared by mixing hollow silica particles and cellulose fibers was treated with hydrophobic silane vapors in order to get a hydrophobic coating. A water droplet was placed on the coated composite. Then the water droplet was removed and the composite was rubbed for 1-2 minutes with a rugged paper (shown in figure S1). Again a water droplet was placed on the this composite. It was observed that the water droplet remained intact on the composite both (1) before and (2) after rubbing with cloth. Figure S1. shows the rugged paper, composite with water droplet before (a) and after rubbing with cloth (2).

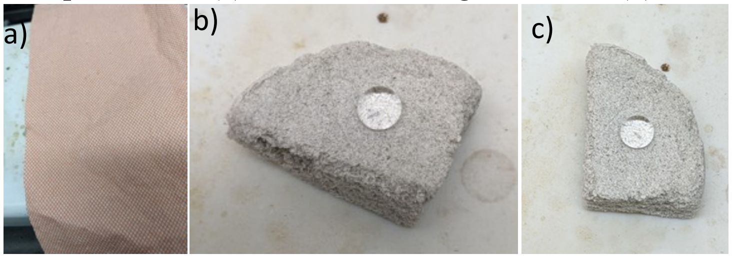

Figure S1. Hydrophobic coating adhesion test. (a) rugged paper used to rub the composite, (b) composite with water droplet before, (c) composite with water droplet after rubbing with cloth.

Thermal conductivity measurements:

Thermal conductivity measurements were performed using a Transient Plane Source (TPS 2500S) instrument and sensor C5501 (Hot Disc, Goteborg, Sweden). Thermal conductivity measurement scan was made by using heating power 20mW for 10 seconds. All measurements were done on loose particle powders or composite samples without additional compacting or weight at room temperature (22 ºC) and relative humidity ≈ 40. For HSP thermal conductivity measurements, the sensor (5501) was sandwiched in between the particle powder bed by using the manufacturer provided sample holder. Figure S2 (left) shows the setup used for measuring the particles thermal conductivity. For measuring the thermal conductivity of the composite, two similar sized pieces of the composite were made, and the sensor was sandwiched between these two pieces. Figure S2 (right) shows the setup used for measuring the thermal conductivity of the composite.

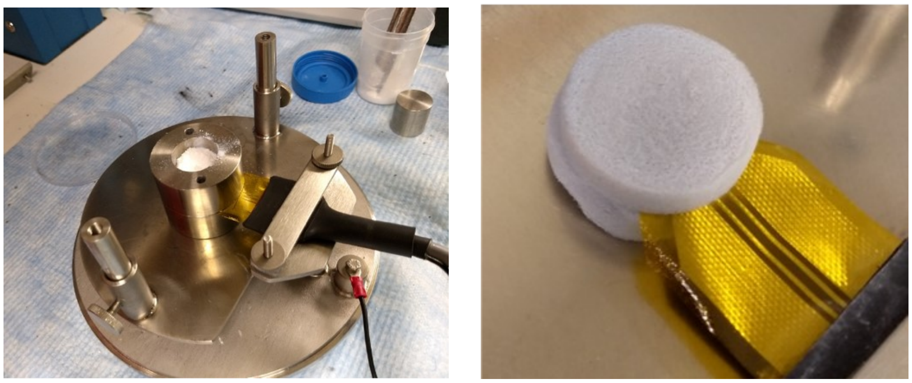

Figure S2. A representative image showing thermal conductivity measurement of HSP powder filled in the sample holder (left) and of the composite pieces sandwiching the sensor (right).

Table 1. Effect of weight on the thermal conductivity measurements of composite and hollow particles:

Weight	Thermal conductivity of Hollow particles	Thermal conductivity of Composite
No weight	0.024 ±0.003 W/m•K	0.020 ±0.002 W/m•K
50g	0.028 ±0.002 W/m•K	0.025 ±0.003 W/m•K
100g	0.032 ±0.004 W/m•K	0.027 ±0.003 W/m•K

It is clear that an increase in weight results in compacting of both HSPs bed and of the composite, and thus results in increasing the thermal conductivity of both materials.

Additional high resolution images of the composite material:

Large pores (≈100 µm) created from ice sublimation

Composite wall made of cellulose fibers, HSPs, and small amount of CB

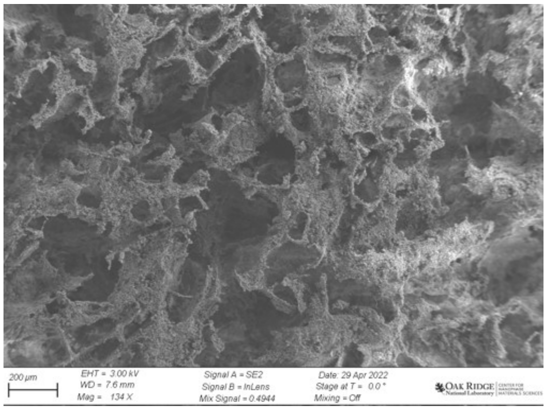

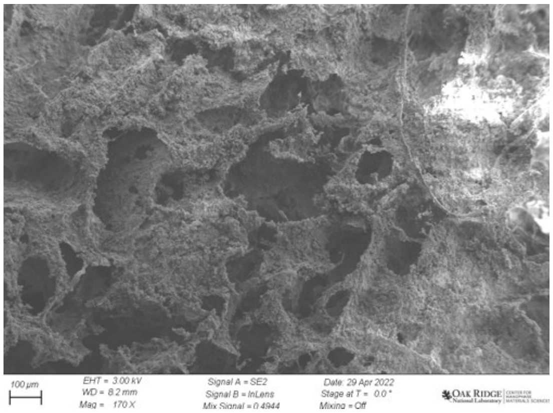

More images of composite wall

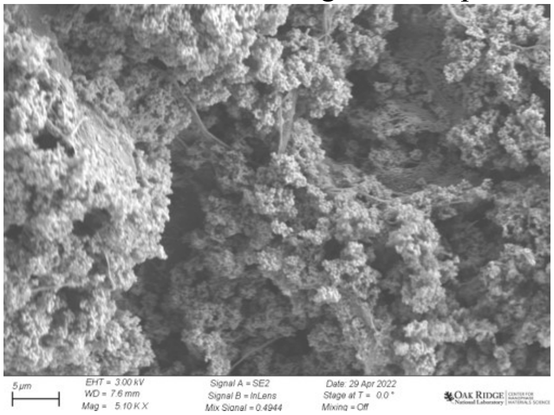

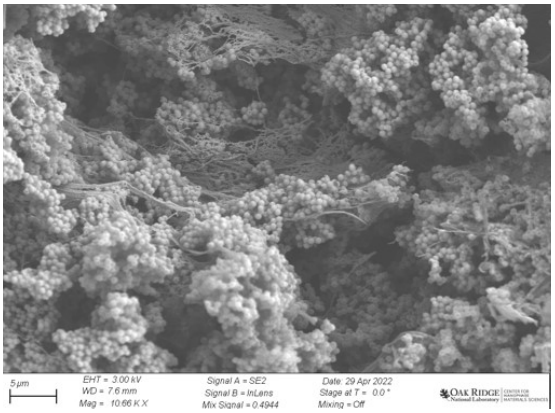

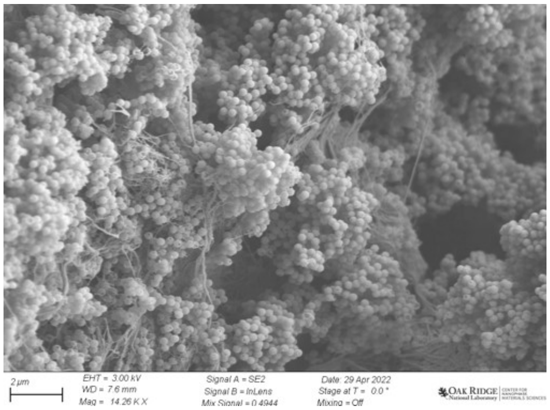

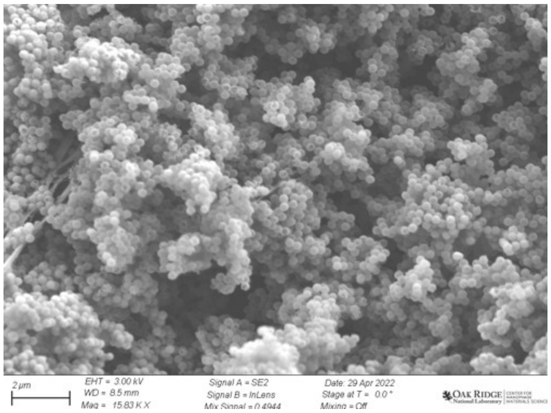

References:

1 J. Sharma, G. Polizos, D. Hun, K. Nawaz and R. Sahore, *RSC Adv.,* 2020, **10**, 31065-31069.
2 D. Spence, D. A. Cullen, G. Polizos, N. Muralidharan and J. Sharma, *Nanomaterials,* 2021, 11, 1627.
